# Dieckol-Attenuated High-Fat Diet Induced Muscle Atrophy by Modulating Muscular Deposition of Lipid Droplets

**DOI:** 10.3390/nu13093160

**Published:** 2021-09-10

**Authors:** Kyung-A Byun, Seyeon Oh, Myeongjoo Son, Seung Eon Oh, Chul-Hyun Park, Kuk Hui Son, Kyunghee Byun

**Affiliations:** 1 Department of Anatomy&Cell Biology, Gachon University College of Medicine, Incheon 21936, Korea; kabyun95@gmail.com (K.-A.B.); mjson@gachon.ac.kr (M.S.); 2 Functional Cellular Networks Laboratory, Department of Medicine, Graduate School and Lee Gil Ya Cancer and Diabetes Institute, College of Medicine, Gachon University, Incheon 21999, Korea; seyeon8965@gmail.com (S.O.); np0520@naver.com (S.E.O.); 3 Department of Thoracic and Cardiovascular Surgery, Gachon University Gil Medical Center, Gachon University, Incheon 21565, Korea; cdgpch@gilhospital.com

**Keywords:** perilipin2, lipid droplet, lipotoxicity, muscle atrophy

## Abstract

An excessive fat diet induces intramuscular fat deposition that accumulates as a form of lipid droplet (LD) and leads to lipotoxicity, including muscle atrophy or decreasing muscle strength. Lipotoxicity depends on the number of LDs, subcellular distribution (intermyofibrillar, IMF, LDs or subsarcolemmal, SS), and fiber type-specific differences (type I or type II fiber) as well as the size of LD. *Ecklonia cava* extracts (ECE), which is known to increase peroxisome proliferator-activated receptor alpha (PPAR-α), which leads to decreasing expression level of perilipin2 (PLIN2). PLIN2 is involved in modulating the size of LDs. This study shows that ECE and dieckol could decrease PLIN2 expression and decrease the size and number of LDs in the muscle of high-fat diet (HF)-fed animals and lead to attenuating muscle atrophy. Expression level of PPAR-α was decreased, and PLIN2 was increased by HF. ECE and dieckol increased PPAR-α expression and decreased PLIN2. The diameter of LDs was increased in high-fat diet condition, and it was decreased by ECE or dieckol treatment. The number of LDs in type II fibers/total LDs was increased by HF and it was decreased by ECE or dieckol. The SS LDs were increased, and IMF LDs were decreased by HF. ECE or dieckol decreased SS LDs and increased IMF LDs. The ECE or dieckol attenuated the upregulation of muscle atrophy-related genes including Murf1, Atrogin-1, and p53 by HF. ECE or dieckol increased the cross-sectional area of the muscle fibers and grip strength, which were decreased by HF. In conclusion, ECE or dieckol decreased the size of LDs and modulated the contribution of LDs to less toxic ones by decreasing PLIN2 expression and thus attenuated muscle atrophy and strength, which were induced by HF.

## 1. Introduction

Intramyocellular lipid (IMCL), which refers to the accumulation of toxic lipids in the myocytes, arises from the increasing uptake of fatty acids (FA) and decreasing removal of FA by decreasing FA oxidation [1]. IMCL is known to be associated with the development of insulin resistance [2,3].

FAs are transported from the plasma to the skeletal muscle fibers by various proteins including FA-binding proteins and FA transport proteins [4]. CD36, or FA translocase, participates in almost 70% of the total FA uptake [5]. The expression of CD36 is increased by high-lipid diet feeding and leads to increasing FA uptake in the skeletal muscle [6].

Fat accumulates as a form of lipid droplets (LDs) in the skeletal muscle. LD is a dynamic organelle formed from a phospholipid monolayer that encloses a core of lipids like triacylglycerols (TGs) [7]. Not all LDs in the muscle are related to lipotoxicity, since LDs act like fuel storage and provide energy source for the muscle [7]. In the healthy muscle, the size of LDs is about 500 nm (range 200–1400 nm) [8]. Normally, type I muscle fibers show greater volume of LDs, mitochondria, and high contact rate between LD and mitochondria compared with type II fibers [9]. The “athlete’s paradox” means trained individuals show higher numbers and smaller LD in type I muscle fibers, whereas patients with type 2 diabetes show bigger LDs in type II fibers [10].

A family known as the PAT (perilipin/adipose differentiation-related protein/tail-interacting protein of 47 kDa) proteins exists in the surface of LD [7]. The PLIN family has five members: PLIN1 to PLIN5.

PLIN2, PLIN3, and PLIN5 are mainly expressed in the human skeletal muscle [11]. PLIN plays an important role in lipolysis regulation, by recruiting or inhibiting the entrance of lipases including adipose triglyceride lipase (ATGL) and hormone-sensitive lipase into LDs [12,13]. PLIN is also involved in regulating energy homeostasis [14]. PLIN2 is involved in increasing the size of LDs [15] and reducing lipolysis [16]. Overexpression of PLIN2 enhances LD accumulation, though PLIN2 knockdown decreases the LD formation and enhances fat oxidation in myotubes [17]. Obesity with insulin resistance causes fatty liver disease [18,19]. The association between PLIN2 and IMCL induced by excessive calorie intake results in increasing volume of adipose tissue, lipid storage, and abnormal LD deposition in non-adipose tissue, which leads to lipotoxicity [17]. PLIN2 is known to be associated with the development of high-fat diet (HF)-induced obesity [20]. PLIN2 deletion prevents weight gain or obesity by HF [17]. Additionally, PLIN2 knockdown leads to decreasing TG levels, increasing insulin sensitivity, and resistance to fatty liver disease [17]. Other studies showed that increased PLIN2-related expression was investigated in the development of sarcopenia as well as metabolic disease induced by lipotoxicity [20]. Intramuscular TG (IMTG) and PLIN2 expression in the muscle of an elderly was increased, and those are negatively associated with quadriceps strength [21]. Moreover, PLIN2 expression showed positive association with muscle atrophy-related genes, including muscle RING-finger protein-1 (Murf1), Atrogin-1, and p53, which suggests that PLIN2 is involved in muscle aging and atrophy [21]. After muscle atrophy was induced by denervation of the tibialis anterior muscle of mice, PLIN2 expression was significantly increased [22]. These results showed the association between PLIN2 and muscle atrophy.

Peroxisome proliferator-activated receptor alpha (PPAR-α) is a nuclear receptor that is mainly present in tissues that show high amount of FA oxidation such as liver and muscle [23]. It controls target genes related to the transportation and oxidation of FA [24]. Phlorotannins extracts from *Ecklonia cava* have anti-obesity effects by increasing activation of AMP protein kinase (AMPK) in the HF-fed obesity animal model [25]. PPAR-α expression is upregulated by AMPK in the muscle [26]. HF for 4 weeks was reported to induce PPAR-α downregulation in mouse liver, and dieckol, one of phlorotannins, restored PPAR-α expression [27]. PPAR-α upregulation was shown to induce decreasing expression level of PLIN2 and lead to decreased lipid storage in the hepatocyte-like cells differentiated from human pluripotent stem cells, which were cultured with oleic acid to mimic nonalcoholic liver disease [28].

Even though dieckol is known to increase PPAR-α and PPAR-α decreased PLIN2, whether dieckol-attenuated HF induced muscle atrophy by modulating deposition of LDs in the muscle by decreasing PLIN2 expression has not been revealed. Here, the hypothesis was that *Ecklonia cava* extracts (ECE) and dieckol could decrease PLIN2 expression and the size and number of LDs, especially in the type II muscle fibers of HF-fed animals, and lead to attenuating muscle atrophy.

## 2. Materials and Methods

### 2.1. HF Animal Model and Material Preparation

C57BL/6N male mice (7-week-old) were bought from Orient Bio (Sungnam, Korea) and were cared for in a controlled temperature (23–24 °C with 50 percent humidity) under a light/dark cycle (12 h/12 h). After 7 days of the adaptation period, the mice were randomly divided into six groups, as follows; Group 1 was fed with a chow diet for 8 weeks. Oral administration of 0.9% normal saline proceeded to mice with a chow diet for the last 4 weeks (group 1, *n* = 6). Groups 2 to 6 were fed with 45 percent HF (Research Diet, Inc., New Brunswick, NJ, USA) for 8 weeks. For the last 4 weeks, the mice were administered with 0.9% normal saline (group 2, *n* = 6), ECE (group 3: 50 mg/kg/day, *n* = 6; group 4: 100 mg/kg/day, *n* = 6; and group 5: 150 mg/kg/day, *n* = 6), or dieckol (group 6: 2.5 mg/kg/day, *n* = 6) by oral gavage, which is an isolation method described in a previous study [29]. After 8 weeks, the bodyweight, fat mass, and lean mass of mice were measured, and then, muscles of the mice were collected following the ethical principles of the Institutional Animal Care and Use Committee of Gachon University (approval number: LCDI-2019-0130).

### 2.2. Isolation of Protein and Western Blotting

The frozen muscle tissues (50 mg) were lysed using 300 µL of RIPA buffer (EzRIPA, ATTO, Tokyo, Japan) with proteinase and phosphatase inhibitors. The homogenized muscle tissues were sonicated and then centrifuged at 14,000× *g* for 15 min at 4 °C. The supernatants were carried on cleaned tubes. The total protein concentration of isolated protein was determined by a bicinchoninic acid assay kit (BCA kit; Thermo Fisher Scientific, Inc., Waltham, MA, USA). The isolated proteins samples were separated on 8 or 10 percent sodium dodecyl sulfate polyacrylamide gel electrophoresis and transferred to polyvinylidene fluoride membrane using a power station (WSE-3500, ATTO, Tokyo, Japan). Then, the membranes were blocked with 5% skim milk (SKI500, LPS solution, Daejeon, Korea) in tris buffered saline containing 0.1% tween-20 (TTBS) for 1 h at room temperature. After washing with TTBS, the membranes were incubated with diluted primary antibodies (Table S1). The probed membranes were rinsed with TTBS and loaded with appropriate peroxidase secondary antibodies. After a final wash with TTBS, the membranes were exposed with an enhanced chemiluminescence kit (GE Healthcare, Chicago, IL, USA) by LAS-4000s (GE Healthcare, Chicago, IL, USA).

### 2.3. Combined Immunofluorescence and Oil Red O Staining

The muscle tissues were cryosectioned at 10 µm. Tissues were washed with phosphate-buffered saline (PBS). The washed tissues were incubated with primary antibody (list are detailed in Table S2) at 4 °C for 12 h and were then rinsed with PBS. Then, the slides were loaded with appropriate fluorescence-conjugated secondary antibody (Alexa Fluor 488; Invitrogen, Waltham, MA, USA) for 1 h at room temperature and then rinsed with PBS. After being washed with PBS, tissues were stained with oil red O working solution for 30 min at room temperature. The detailed method of oil red O staining was described previously [30]. After oil red O staining, the tissue was incubated with 4′,6-diamidino-2-phenylindole (Sigma-Aldrich, St. Louis, MO, USA) solution for 10 s at room temperature, washed with PBS, and mounted using vector shield solution (Vector Laboratories, Burlingame, CA, USA). The signal of fluorescence was identified by a confocal microscope (LSM 710, Carl Zeiss, Oberkochen, Germany).

### 2.4. Extraction of RNA and Quantitative Real-Time-Polymerase Chain Reaction (qRT-PCR)

The frozen muscle was lysed with 500 µL of RNiso (Takara, Shiga, Japan) using homogenizer. After mixing with chloroform, the samples were centrifuged at 12,000× *g* for 15 min at 4 °C. The transparent layers were collected in cleaned tubes, mixed with the same amount of isopropanol as the aqueous layer, and then centrifuged at 12,000× *g* for 15 min at 4 °C. Isolated RNA samples were washed with 75 percent ethanol and dried. Then, RNA pellet was dissolved with diethyl pyrocarbonate-treated water. The cDNA was synthesized using a PrimeScript First Strand cDNA Synthesis Kit (Takara, Shiga, Japan). The qRT-PCR was conducted using the CFX 384 Touch™ Real-Time PCR Detection System (Bio-Rad Laboratories, Irvine, CA, USA). The reaction efficiency and cycle threshold values were identified by the CFX Manager™ software (Bio-Rad Laboratories, Irvine, CA, USA). For internal control, *actb* was used, and the primer sequences for the target genes are listed in Table S2.

### 2.5. Hematoxylin and Eosin (H&E) Staining

The muscle paraffin tissues were deparaffinated and rehydrated. The nucleus of cells was stained with hematoxylin (DAKO, Glostrup, Denmark) for 30 s and cytosol of cells was stained with eosin (Sigma-Aldrich, St. Louis, MO, USA) for 30 s. H&E-stained sections were visualized using light microscopy (Olympus Optical Co., Tokyo, Japan), and quantification of the cross-sectional area size was measured using ImageJ software (National Institutes of Health, Bethesda, MD, USA).

### 2.6. Grip Strength

A grip strength meter (JD-A-22, JEUNGDO BIO& PLANT CO. LTD, Seoul, Korea) measure the mouse or rat grip strength. The grip strength meter digitally displays the maximum force. After the mouse was placed on the meter bar, the tail of mouse was carefully pulled. Ten measurements were performed at one-minute intervals and used as the average.

### 2.7. Statistical Analysis

In this study, the non-parametric tests were used. To identify the significance of differences among the six groups, the Kruskal–Wallis test was conducted, and if a significant difference was confirmed, Mann–Whitney U test was used for multiple comparisons. All experiments conducted in this study were performed three times per mice, and the graph value of results were indicated as the mean ± standard deviation (SD). The statistical analysis was analyzed by SPSS version 22 (IBM Co., Armonk, NY, USA) and symbols of all data were used *, $, and # (*, vs. Chow/Saline; $, vs. HF/Saline; #, vs. HF/ECE100).

## 3. Results

### 3.1. ECE and Dieckol Decreased the Expressions of CD36 and PLIN2 and Increased PPAR-α in the Muscles of HF-Fed Animals

**Figure 1:**
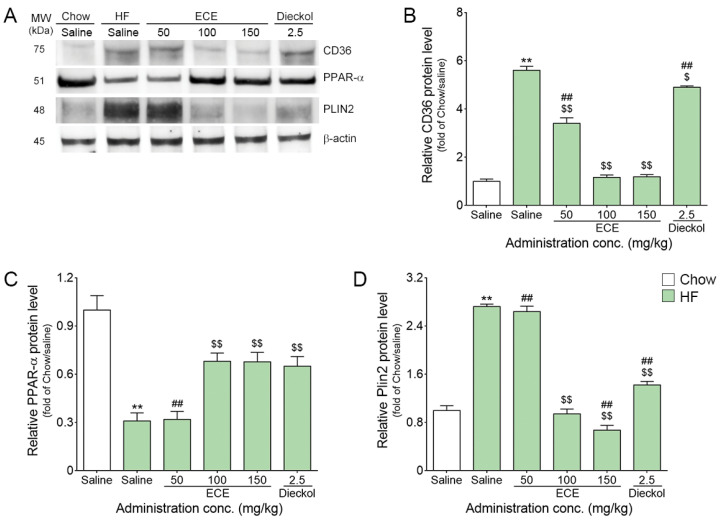
Effects of ECE and dieckol on the CD36, PPAR-α, and PLIN2 modulation in the muscle of HF-fed mice. (**A**) The protein levels of CD36, PPAR-α, PLIN2 and β-actin were determined by western blotting. The β-actin was used for comparison for equal amount of protein level, and relative comparisons were mad for the Chow/saline group. (**B**) The CD36 protein level of the HF/saline groups were increased and decreased by ECE or dieckol treatment. (**C**) The PPAR-α protein level of the HF/saline groups were decreased and increased by ECE or dieckol treatment. (**D**) The PLIN2 protein level of the HF/saline groups was increased and decreased by ECE or dieckol treatment. Data are presented as mean ± SD. **, *p* < 0.01 vs. Chow/saline; $, *p* < 0.05 and $$, *p* < 0.01 vs. HF/saline; ##, *p* < 0.01 vs. HF/ECE100 (Mann–Whitney U test). CD36, cluster of differentiation 36; conc., concentration; ECE, *Ecklonia cava* extract; HF, high-fat diet; PLIN2, Perilipin-2; PPAR-α, peroxisome proliferator-activated receptor alpha.

The expression level of CD36 in the muscle was significantly increased by HF, and it was significantly decreased by the ECE or dieckol treatment (Figure 1A,B and Figure S1A). The decreasing effect was most prominent at 100 mg/kg ECE and 150 mg/kg ECE.

The expression level of PPAR-α in the muscle was significantly decreased by HF, and it was significantly increased by the 100 mg/kg ECE, 150 mg/kg ECE and dieckol treatment (Figure 1A,C and Figure S1B). The increasing effect was most prominent at 100 mg/kg ECE, 150 mg/kg ECE and dieckol.

The expression level of PLIN2 in the muscle was significantly increased by HF, and it was significantly decreased by the 100 mg/kg ECE, 150 mg/kg ECE and dieckol treatment (Figure 1A,D and Figure S1C). The decreasing effect was most prominent at 150 mg/kg ECE.

### 3.2. ECE and Dieckol Decreased the Size and Number of LDs and Increased the Deposit of LD Near the Mitochondria in the Muscle of HF-Fed Animals

**Figure 2:**
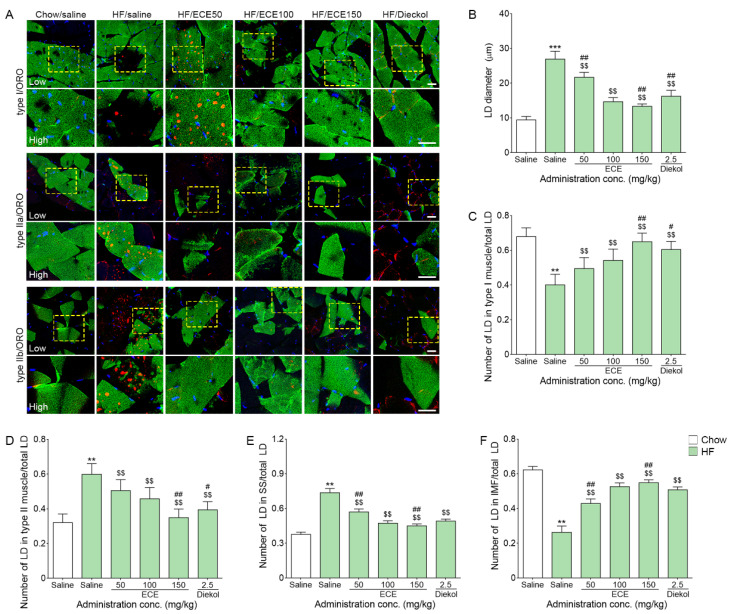
Effects of ECE and dieckol on lipid droplets (LDs) modulation in the muscle. (**A**) The images of low and high magnification show the myosin type I, IIa or IIb with oil red O staining. (**B**) The average size of LDs in the muscle was increased by HF/saline and decreased after treatment with ECE or dieckol. (**C**) The numbers of LDs in type I fiber were increased by ECE or dieckol treatment. (**D**) The numbers of LDs in type II fiber were increased by HF/saline and were decreased after treatment with ECE or dieckol. (**E**) The number of LDs in the SS muscle was increased by HF/saline and decreased after treatment with ECE or dieckol. (**F**) The number of LDs in the IMF muscle was decreased by HF/saline and increased after treatment with ECE or dieckol. Scale bar = 100 µm. Data are presented as mean ± SD. **, *p* < 0.01 and ***, *p* < 0.001 vs. Chow/saline; $$, *p* < 0.01 vs. HF/saline; #, *p* < 0.05 and ##, *p* < 0.01 vs. HF/ECE100 (Mann–Whitney U test). conc., concentration; ECE, *Ecklonia cava* extract; HF, high-fat diet; IMF, intermyofibrillar; SS, subsarcolemmal.

The average size of LDs, which was measured as droplet diameter, was significantly increased in the muscle by HF, and it was significantly decreased by the ECE or dieckol treatment (Figure 2A,B). The most prominent decreasing effect was shown at 150 mg/kg ECE.

The ratio of numbers of LDs that deposit on the type I fibers to total LDs (the number of LD in type I fibers/total LDs) were significantly decreased by HF, and it was significantly increased by the ECE or dieckol treatment (Figure 2C). The increasing effect was most prominent at 150 mg/kg ECE.

The number of LDs in type II was the sum of the number of LD deposit on the type IIa and type IIb. The LD in type II/total LDs was significantly increased by HF, and it was significantly decreased by the ECE or dieckol treatment (Figure 2D). The most prominent decreasing effect was shown at 150 mg/kg ECE.

The number of LDs was also measured depending on the subcellular distribution of intermyofibrillar (IMF) LDs or subsarcolemmal (SS). The ratio of the number of SS LDs to total LDs was increased by HF, and it was significantly decreased by the 150 mg/kg of ECE treatment (Figure 2E).

The ratio of the number of IMF LDs to total LDs was decreased by HF, and it was significantly increased by the ECE ordieckol treatment (Figure 2F). The most prominent increasing effect was shown at 150 mg/kg ECE.

### 3.3. ECE and Dieckol Attenuated the Expression of Murf1, Atrogin-1, and p53 in the Muscle of HF-Fed Animals

**Figure 3:**
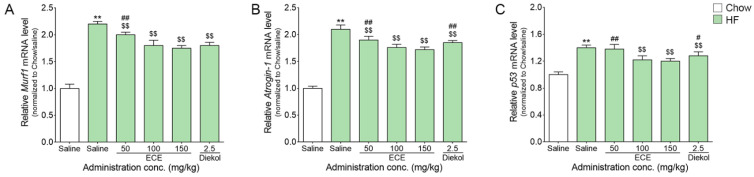
Effects of ECE and dieckol on the *Murf1*, *Atrogin-1*, and *p53* modulation in the muscle of HF-fed mice. (**A**–**C**) The *Murf1* (**A**), *Atrogin-1* (**B**), and *p53* (**C**) mRNA levels of the HF/saline groups were increased and decreased by ECE or dieckol treatment. Data are presented as mean ± SD. **, *p* < 0.01 vs. Chow/saline; $$, *p* < 0.01 vs. HF/saline; #, *p* < 0.05 and ##, *p* < 0.01 vs. HF/ECE100 (Mann–Whitney U test). conc., concentration; ECE, *Ecklonia cava* extract; HF, high-fat diet; Murf1, muscle RING-finger protein-1; p53, tumor protein P53.

The expression level of *Murf1* was significantly increased by HF, and it was significantly decreased by the ECE or dieckol treatment (Figure 3A). The most prominent decreasing effect was shown at 100 mg/kg ECE, 150 mg/kg ECE, and dieckol.

The expression level of *Atrogin-1* was significantly increased by HF, and it was significantly decreased by the ECE or dieckol treatment (Figure 3B). The most prominent decreasing effect was shown at 100 mg/kg ECE and 150 mg/kg ECE.

The expression level of *p53* was significantly increased by HF, and it was significantly decreased by the ECE or dieckol treatment (Figure 3C). The most prominent decreasing effect was shown at 100 mg/kg ECE and 150 mg/kg ECE.

### 3.4. ECE and Dieckol-Attenuated Muscle Atrophy and Improved Grip Strength

**Figure 4:**
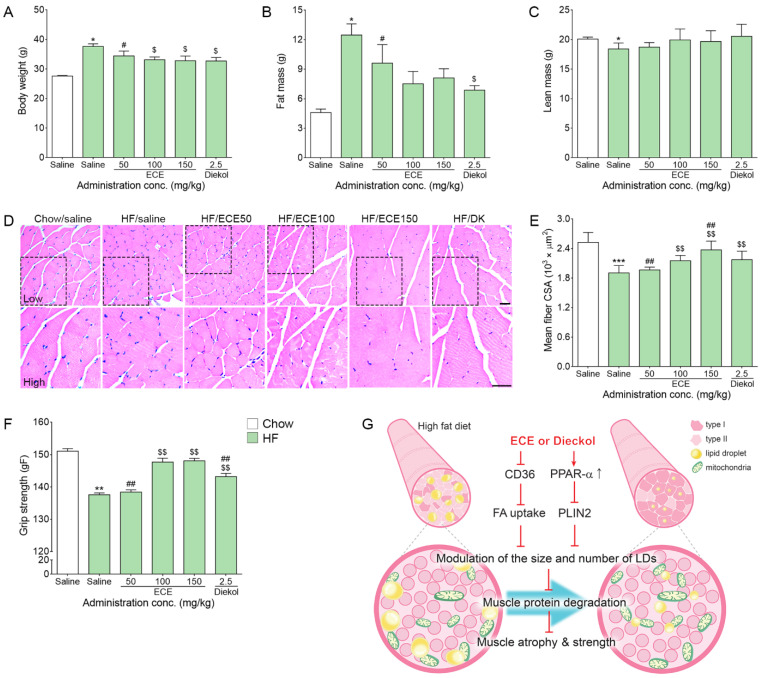
Effects of ECE and dieckol on the weight, muscle atrophy, and grip strength modulation in the livers of HF-fed mice. (**A**) Bodyweight was increased by HF/saline and decreased by ECE or dieckol treatment. (**B**) Fat mass was increased by HF/saline and decreased by dieckol treatment. (**C**) Lean mass was decreased by HF/saline but not increased by ECE or dieckol treatment. (**D**,**E**) The CSA of the muscle fiber by hematoxylin and eosin (H&E) staining was decreased by HF/saline and increased by ECE or dieckol treatment. (**F**) The grip strength was decreased by HF/saline and increased by ECE or dieckol treatment. (**G**) The schematic summary of the effect of ECE or dieckol in high fat diet muscle. Scale bar = 100 µm. Data are presented as mean ± SD. *, *p* < 0.05, **, *p* < 0.01 and ***, *p* < 0.001 vs. Chow/saline; $, *p* < 0.05 and $$, *p* < 0.01 vs. HF/saline; #, *p* < 0.05 and ##, *p* < 0.01 vs. HF/ECE100 (Mann–Whitney U test). conc., concentration; ECE, *Ecklonia cava* extract; HF, high-fat diet.

Body weight was significantly increased by HF, and it was significantly decreased by the ECE or dieckol treatment (Figure 4A). The most prominent decreasing effect was shown at 100 mg/kg ECE, 150 mg/kg ECE, and dieckol.

Fat mass was significantly increased by HF, and it was decreased by the ECE or dieckol treatment (Figure 4B). The most prominent decreasing effect was shown at dieckol. The lean mass was significantly decreased by HF, and it was not significantly decreased by the ECE or dieckol treatment (Figure 4C).

The mean cross-sectional area (CSA) of the muscle fibers was significantly decreased by HF, and it was significantly increased by the 100 mg/kg ECE, 150 mg/kg ECE, and dieckol treatment (Figure 4D,E). The increasing effect was most prominent at 150 mg/kg ECE.

The grip strength was significantly decreased by HF, and it was significantly increased by the 100 mg/kg ECE, 150 mg/kg ECE, and dieckol treatment (Figure 4F). The increasing effect was most prominent 100 mg/kg ECE and 150 mg/kg ECE.

## 4. Discussion

An increased amount of IMCL is associated with insulin resistance in non-obese adults [2] and HF-fed animal models [31]. Paradoxically, the amount of IMCL is increased in trained athletes, even though their insulin sensitivity is high [32]. How IMCL affects lipotoxicity like insulin resistance is not only determined by the size of LDs. The number of LDs, subcellular distribution (IMF or SS), and fiber type-specific differences (type I or type II fiber) could determine the association between IMCL and lipotoxicity [10]. Previously, the size of LD was reported to be negatively associated with insulin sensitivity [33]. However, another study showed that no significant difference was observed between type 2 diabetes and trained athlete in the total content of IMCL, and the insulin sensitivity was more affected by the lipid storage pattern [10]. Type 2 diabetes patients had more IMCL that had a lower number of LDs, but larger-sized LDs located mainly in type II fibers in the SS area, whereas athletes had IMCL that had a higher number of LDs and normal-sized LDs in type I fibers, mainly located in the IMF region [10]. It suggested that large LDs in type II fibers are riskier and more related to lipotoxicity [10]. In the muscle fibers, lipid is stored in either the IMF or SS region. Since LDs are more closely connected to the mitochondria in IMF than are those in the SS region, LDs in SS are more related to the development of lipotoxicity than are LDs in IMF [34,35].

PLIN2 is known to have a role in increasing the size of LDs. PLIN2 overexpression promotes LD accumulation in cultured fibroblasts [36] or human embryonic kidney cells by reducing the connection of ATGL to LDs [37]. PLIN2-knockout mice ameliorate lipid accumulation in the liver and hepatic steatosis [19,38] and formation of macrophage foam cell [39], whereas overexpression of PLIN2 causes severe hepatic steatosis [40]. PLIN2 overexpression enhances the accumulation of intramyocellular LDs and TG storage, whereas PLIN2 knockdown showed opposite effects [17]. It is also reported that knockout PLIN2 animals showed fewer but larger LDs in the skeletal muscle than wild type [17]. Oleic acid induced dramatic upregulation of PLIN2 in C2C12 cells [41].

Here, our study showed that HF induced an increased expression level of CD36 in the skeletal muscle. HF also induced decreased expression of PPAR-α. PPAR-α is known to be related to PLIN2 expression [28]. Our result showed that HF increased PLIN2; however, the ECE or dieckol treatment restored the expression level of PPAR-α and decreased the expression level of PLIN2 in the skeletal muscle.

As a principal translocase, increased CD36 may increase FA uptake in the muscle and increased LD size. Additionally, increased expression level of PLIN2 is also involved in the enlargement of LD size [15,16].

Here, the average size of LDs, which is measured by the diameter of LDs in our study, was significantly increased by HF, and it was significantly decreased by the ECE and dieckol treatment. The number of LDs was also evaluated depending on muscle fiber type and subcellular distribution. The number of LD in type I fibers/total LDs was significantly decreased by HF and it was restored by ECE or dieckol treatment. However, the number of LDs in type II fibers/total LDs was significantly increased by HF and it was decreased by ECE or dieckol. The ratio of SS LDs to total LDs was increased by HF, and it was significantly decreased by the 150 mg/kg ECE treatment. The ratio of IMF LDs to total LDs was decreased by HF, and it was significantly increased by the ECE or dieckol treatment. These results suggested that HF increased LD deposition in type II fibers. In contrast, LD deposition in type I muscle fibers was decreased by HF. HF increased SS LDs; however, deposition of IMF LDs was decreased by HF. It seemed that HF could increase lipotoxicity by increasing SS LDs and decreasing IMF LDs, which act as the energy source. The ECE or dieckol treatment decreased the number of LDs in the type II muscle fiber, which are known to be more associated with lipotoxicity than LDs in the type I muscle fiber. Additionally, ECE or dieckol decreased SS LDs and increased IMF LDs. Thus, ECE or dieckol modulated LD distribution to be less toxic to the muscle fiber.

PLIN2 expression is known to be associated with a pathological condition including aged muscle, which leads to IMTG accumulation and lipotoxicity. In aged muscle, increased expression of PLIN2 was related to p53 activation and decreased muscle mass and strength [21]. Here, our study showed that expressions of Murf1, Atrogin-1, and p53 were increased by HF and those were decreased by ECE or dieckol.

In addition, our study showed that CSA of the muscle fibers was decreased by HF, and it was attenuated by the ECE or dieckol treatment. Muscle strength, which was evaluated by grip strength, was significantly decreased by HF, and it was also restored by the ECE or dieckol treatment. Interestingly, the lean body mass was significantly decreased by HF, whereas the ECE or dieckol treatment has not prominently increased lean body mass. The total muscle mass, which was presented with lean body mass, was not changed; however, the LD distribution depending on muscle type or subcellular distribution was modulated by the ECE or dieckol treatment. Additionally, the ECE or dieckol treatment decreased muscle atrophy related to signaling pathways including Murf1, Atrogin-1, and p53. ECE or dieckol seems to modulate size, distribution depending on muscle type, and subcellular distribution of LDs and thus leads to decrease muscle atrophy and strength, even though lean body mass was not significantly restored by the ECE or dieckol treatment.

In conclusion, ECE or dieckol enhanced PPAR-α expression, which leads to PLIN2 downregulation and decreased CD36 expression, which leads to decreasing FA uptake. With these, the LD size in the muscle was decreased, and the distribution of LDs depending on the muscle type was changed. The number and size of LDs in the type II fibers were decreased, and LDs around SS were decreased, consequently leading to decreasing lipotoxicity and muscle atrophy by decreasing atrophy-related signals.

## Data Availability

All data supporting the conclusions of this article are included in this article.

## Supplementary Materials

The following are available online at https://www.mdpi.com/article/10.3390/nu13093160/s1: Table S1: List of antibodies for immunoblotting, immunohistochemistry and Table S2: List of primers for qRT-PCR, and Figure S1: Modulation effects of ECE and DK on the *Cd36, Ppar-α,* and *Plin2* in the muscle of HF-fed mice.
