# Supplementary material for: Dieckol-Attenuated High-Fat Diet Induced Muscle Atrophy by Modulating Muscular Deposition of Lipid Droplets
Source: Nutrients. 2021 Sep 10;13(9):3160. doi: 10.3390/nu13093160 (PMC8467349; doi:10.3390/nu13093160)
Supplement: Supplementary file 1 [file nutrients-13-03160-s001.zip › nutrients-1333793-supplementary.pdf]

**Table S1. List of antibodies for immunoblotting and immunohistochemistry**

| Antigen (host) | Company | Catalog no. | Dilution rate |
|---|---|---|---|
| CD36 (rabbit) | Abcam | ab124515 | 1:1000 |
| PPAR$\alpha$ (rabbit) | Bioss | bs-3614R | 1:300 |
| PLIN2 (mouse) | Santa cruz | sc-377429 | 1:100 |
| Myosin heavy chain Type I (mouse) | Developmental Studies Hybridoma Bank | BA-D5 | 1:36.4 |
| Myosin heavy chain Type IIA (mouse) | Developmental Studies Hybridoma Bank | SC-71 | 1:85 |
| Myosin heavy chain Type IIB (mouse) | Developmental Studies Hybridoma Bank | BF-F3 | 1:68.4 |

**Table S2. List of primers for qRT-PCR**

| Gene | | Primers |
|---|---|---|
| *Actb* | Forward | 5'-CCGTAAAGACCTCTATGCCAAC-3′ |
| | Reverse | 5'-GCAGTAATCTCCTTCTGCATCC-3′ |
| *Cd36* | Forward | 5'-CAA TGC CTT TGC ATC ACC CC-3′ |
| | Reverse | 5'-GGC CAT CTC TAC CAT GCC AA-3′ |
| *Ppar-α* | Forward | 5'-CTG GTC TTA ACC GGC CCA AT-3′ |
| | Reverse | 5'-TGC ACA TAG CCA GAA GGG TG-3′ |
| *Plin2* | Forward | 5'-TGC TGT GTG GTG ATC TGG AC-3′ |
| | Reverse | 5'-AAC AAT CTC GGA CGT TGG CT-3′ |
| *Murf1* | Forward | 5'-GTG TGA GGT GCC TAC TTG CT-3′ |
| | Reverse | 5'-GAC TTT TCC AGC TGC TCC CT-3′ |
| *Atrogin-1* | Forward | 5'-TGA CAA AGG GCA GCT GGA TT-3′ |
| | Reverse | 5'-AGG GTT TCT CAA TGA CAG CG-3′ |
| *p53* | Forward | 5'-CAG ACT GAC TGC CTC TGC AT-3′ |
| | Reverse | 5'-CTT GGG CCA GGA ACC ACT AC-3′ |

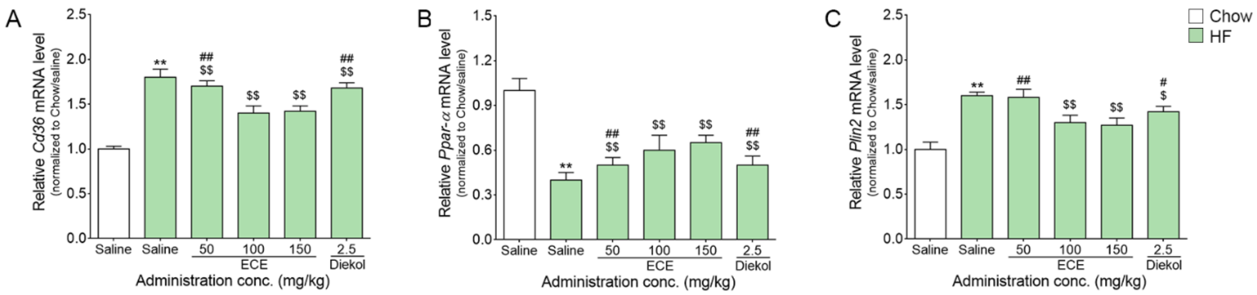


**Figure S1. Modulation effects of ECE and DK on the *Cd36, Ppar-α,* and *Plin2* in the muscle of HF-fed mice.** (A-C) The mRNA levels of *Cd36, Ppar-α,* and *Plin2* were measured by qRT-PCR. Relative comparison of target genes was analyzed for changes in Chow/Saline group after normalization with *Actb* gene. Data are presented as mean ± SD. **, $p < 0.01$ vs. Chow/saline; \$, $p < 0.05$ and \$\$, $p < 0.01$ vs. HF/saline; #, $p < 0.05$ and ##, $p < 0.01$ vs. HF/ECE100 (Mann-Whitney U test). Cd36, cluster of differentiation 36; conc., concentration; ECE, *Ecklonia cava* extract; HF, high-fat diet; Plin2, Perilipin-2; Ppar-α, peroxisome proliferator-activated receptor alpha
